# Effects of riverbank erosion on mental health of the affected people in Bangladesh

**DOI:** 10.1371/journal.pone.0254782

**Published:** 2021-07-22

**Authors:** Altaf Hossain, Md. Jahangir Alam, Md. Rezaul Haque

**Affiliations:** 1 Department of Statistics, Islamic University, Kushtia, Bangladesh; 2 Department of Statistics, University of Rajshahi, Rajshahi, Rajshahi, Bangladesh; Population Council, INDIA

## Abstract

**Background:**

In Bangladesh, riverbank erosion is a major problem that regularly displaces millions of people and affects their mental health every year.

**Objectives:**

The primary objective is to explore the effects of riverbank erosion on mental health problems such as depression, anxiety, and stress in Bangladesh.

**Methods:**

We conducted a household survey from August 2019 to November 2019 on randomly selected adult respondents from Rajbari District located along the Ganges River and Tangail District located along the Brahmaputra River. The respondents were divided into two groups: exposed and non-exposed to riverbank erosion. All participants were asked to complete self-reported questionnaires on the Depression, Anxiety and Stress Scale-21, and other socio-demographic, economic and riverbanks erosion-related factors. We performed Chi-squared test and multiple logistic regression analysis to explore the significant risk factors (*P*<0.05) of mental illness (depression, anxiety and stress).

**Results:**

We surveyed 611 households, of whom 410 were from Rajbari and 201 were from Tangail. Among 611 respondents, 509 (83.31%) were exposed by riverbank erosion whereas 102 (16.69%) were non-exposed. The prevalence of depression, anxiety and stress (DAS) was 38.30%, 76.60%, 32.41%, respectively, and they were significantly higher among the exposed group than the non-exposed group (depression: 45.19% versus 3.92%, *P*<0.001; anxiety: 82.71% versus 46.08%, *P*<0.001; stress: 38.11% versus 3.92%, *P*<0.001). The respondents exposed to river erosion were respectively 8.28, 2.26 and 5.09 times more likely to develop DAS disorder compared to their non-exposed counterparts (OR_D_ = 8.28, 95% CI = 2.75–24.89; OR_A_ = 2.26, 95% CI = 1.31–3.88; OR_S_ = 5.09, 95% CI = 1.64–15.76). Females and those who lost their houses and displaced, were more likely to have DAS disorder compared to their respective counterparts.

**Conclusions:**

The exposed people were more likely to experience mental health problem and demand some social safety net programs with special focus on female and those who lost houses and displaced.

## Introduction

In the world, around 450 million people are suffering from mental health problems, placing mental disorders among the leading causes of illness and disability. More than 264 million people of all ages suffer from a depressive disorder [1]. Among them, more than 150 million people were adults. The WHO also reported that women are more affected by depression than men. Moreover, around 272.5 million people suffer from anxiety disorder worldwide [2]. It is predicted that one in four people in the globe will be affected by psychological issues or neurological disorders at some point in their life [1]. About 50% of people who committed suicide had depression or another mood of disorder [3]. Natural disasters affect millions of people psychologically and physically all over the world [4], and the consequences are widespread and maybe long term. Riverbank erosion is such a natural disaster, which has been linked to increased prevalence of mental disorders such as depression, anxiety, stress, in both developed and developing countries. Around 30–50% of the natural disaster-affected people are related to moderate to severe psychological distresses [5, 6]. Among the disaster victims, depression, anxiety, stress, major depressive disorder (MDD), post-traumatic stress disorder (PTSD), phobia, behavioral problems, and prolonged grief are common [4, 7]. Depression, anxiety, and tension/stress are now the major health problems that cause disability globally, and no one immune to these problems [8–10].

In Bangladesh, the prevalence of mental health is more serious than in other South Asian countries [11]. Nearly 17% of adults in Bangladesh are suffering from different mental health problems with 16.8% men and 17% women, and among them, 32.3% don’t seek medical attention [12]. In 2005, a survey conducted in Bangladesh revealed that 16.1% of the adult population suffered from some kind of mental disorder in Bangladesh. The survey shows, 6.7% have a depressive disorder, 4.5% anxiety, and 2.1% somatic symptoms and related disorders [12].Again, climate change exposes humans to more frequent and higher intensity of extreme weather events (e.g., riverbank erosion) than other natural variability, and disrupts ecosystems, economies and social systems [13–19]. In Bangladesh, 24 out of 64 districts are classified as climate-displacement-prone [20]. Half of these districts are located in the riverbank erosion, flood, and displacement-prone areas, where about 500,000 people annually experience displacement [21]. The number of households affected by natural disasters in Bangladesh has increased from 550,555 in 2009 to 1,934,629 in 2014 [22]. Around 2,270, hectares of land was lost due to riverbank erosion, and at least 5,081 families were displaced in 2019 [23, 24]. Khatun et al. [25] anticipated if the trend of increasing frequency and intensity of natural disasters continues, about 30,366,230 households in Bangladesh will be affected by natural disasters in 2030. The effects of those extreme weather events not only contribute to migration/displacement but also influence the proximate determinants of both physical and mental health [17, 26].

Among the different types of natural disasters, riverbank erosion is less discussed in the social, political, or scientific community though it is happening most of the time somewhere in the world. Moreover, there are very limited studies worldwide where the effect of river erosion and its associated factors on mental health (depression, anxiety and stress) is well studied. To the best of our knowledge, there is no research study that explored the effect of river erosion and its associated factors on mental health status (depression, anxiety and stress) in Bangladesh. In this study, we determined the effect of river erosion and its related vital risk factors on mental health status in Bangladesh.

## Materials and methods

### The study location/area

We purposively selected Rajbari and Tangail Districts as our study areas because those areas are strikingly prone to river erosion. The selection of the study area is mainly based on the following considerations: (i) people affected by river erosion are available in the area, (ii) erosion of the areas is recent and ongoing, (iii) the villages of these districts are easily accessible for the survey, and (iv) high expectation of cooperation and obtaining reliable data from the respondents, the possibility of cooperation and getting reliable data from the respondents were expected to be high. A total of 14 villages were selected: 4 from Arjuna Union in Tangail District and 10 from Mijanpur and Barat Unions of Rajbari District. Those villages were on landmasses in the basins of the Brahmaputra and Ganges rivers.

**Fig 1:**
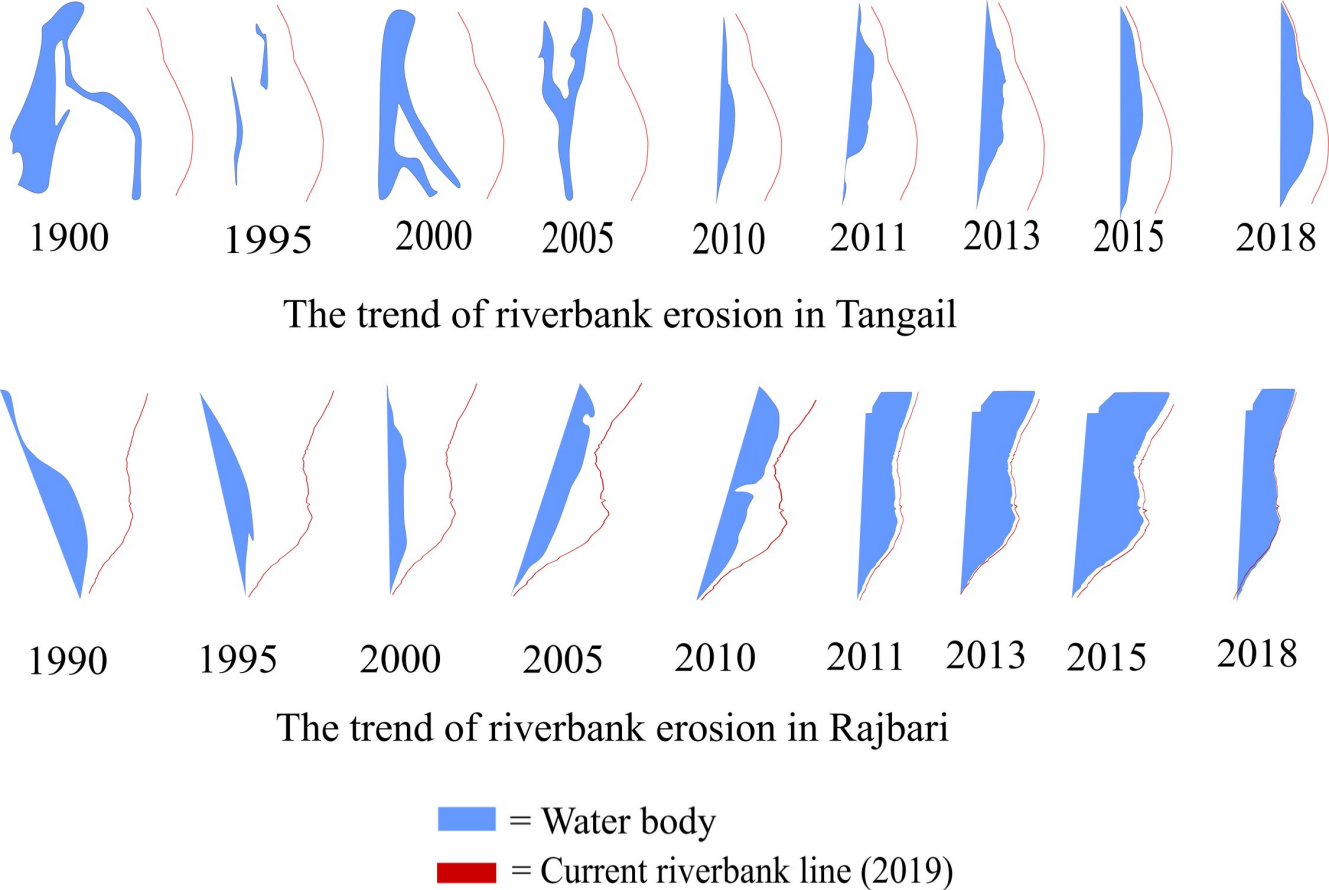
The trend of riverbank erosion of the study area.

To understand the historic trend of riverbank erosion, we traced the current river bank line by walking along the study area with the Vespucci application in an android phone; the line of Tangail was drawn on 30 November 2019 and the Rajbari on 1 December 2019. The dry season satellite images from 1990 to 2018 were taken and compared with the current river bank line (Fig 1).

From the graph, we observed that almost 254 hectares of land were grabbed by the erosion of the left bank of the Brahmaputra River in the last year along the study area of Tangail District. It was also seen that the river had gradually shifted from right to left in the area. In the Rajbari District, there was no recent severe erosion along the right bank of the Ganges River. Massive riverbank erosion along the study area of the Rajbari District is relatively older as the rate of erosion seems to be slower due to some recent protective measures in the name of protecting Rajbari Town. The government has taken several projects such as providing geotextile sandbags, concrete cement blocks to protect the right bank of the river, and the process is still ongoing. However, the local people do not have confidence in the sustainability of the measures taken by the government. Consequently, they are still worried about their future and carry the lasting and devastating effects of erosion, at least in mind.

### Sampling method

First, we designed a questionnaire including questions on riverbank erosion, socio-economic, and demographic information about the population, along with a well-validated and reliable “Depression Anxiety Stress Scales (DASS-21)” to measure the mental health condition. Then we conducted a cross-sectional household survey in the selected study areas from August 2019 to November 2019 to collect data based on our designed questionnaire (see supplementary appendix S1 Appendix). We first purposively selected two river erosion-prone subdistricts: (i) Rajbari Sadar of Rajbari District and (ii) Bhuyapur of Tangail District as our study area. After this, we purposively selected two Unions (Mijanpur and Barat) from Rajbari Sadar subdistrict and one Union (Arjuna) from Bhuyapur subdistrict. Then we randomly selected eight villages (Ramcondropur, Charjoukuri, Boro-Charbeninagar, Ramkrisnopur, Charnarayanpur, Chilimpur, Dhunchi, Sonakandor), two villages (Vhobodiya and Gopalbari) and four villages (Arjuna, Kuthiboira, Dhubliya and Tarai), respectively, from Mijanpur Union, Barat Union and Arjuna Union, by using the cluster sampling method.

**Fig 2:**
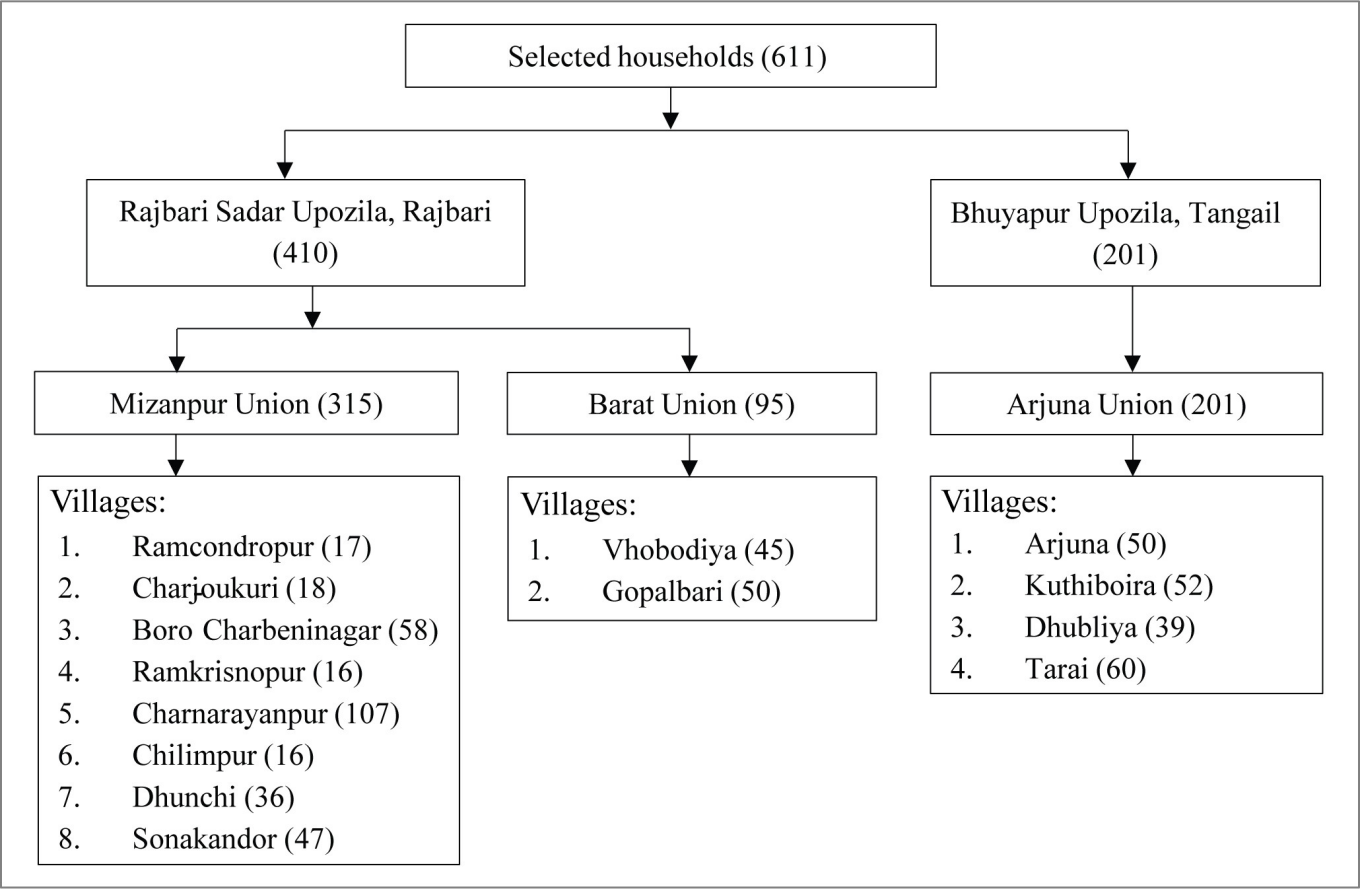
Distribution of the sampled households.

For sample size determination, we used the formula for simple random sampling design as *n* = [*Z*^2^*P*(1−*P*)]/*d*^2^, where *n* = sample size, *Z* = 1.96 (upper limit of 95% confidence interval), *P* = prevalence of mental disorder and *d* = precision [27–30]. According to Haque et al. [27], considering *P* = 0.36, *d* = 0.05, design effects of 1.5 and a 10% non-response rate, a sample size of 590 was estimated to be sufficient for the purposes of statistical analysis. Finally, probability proportional to size (PPS) sampling was used to select the households from each of the selected villages. We selected a sample of 611 households, of which 410 households were selected from the Rajbari District (Mijanpur Union: 315 households and Barat Union: 95 households) and 201 households were selected from the Arjuna Union of Tangail District. The distributions of selected households are given in Fig 2. We interviewed the head of each household, and, in absence of the head, we interviewed the representative person of the household.

### Study variables

#### Outcome variables

In our study, the outcome variables of interest are the three measures of mental health: depression, anxiety and stress status among the study population.

***Depression***: Depression manifests as sadness, feelings, loss of interest or pleasure, disturbed sleep or appetite, guilt or low self-worth, poor concentration, and extreme tiredness [31]. Depression may include a depressed mood, reduced interest or pleasure in activities once enjoyed, a loss of sexual desire, changes in appetite, unintentional weight loss or gain, sleeping too much or too little, agitation, restlessness, and pacing up and down, slowed movement and speech, loss of energy, feelings of worthlessness or guilt, difficulty in thinking, concentrating or making decisions, and recurrent thoughts of death or suicide, or an attempt at suicide [32]. Factors that are likely to play a role to trigger depression include (i) genetic factors (e.g., change in genetic features, personal or family history of depression), (ii) biological factors (e.g., changes in the brain’s neurotransmitter levels, conditions such as bipolar disorder), (iii) environmental factors (such as riverbank erosion, flooding, cyclone), (iv) socio-demographic factors (e.g., age, education, employment), and (v) psychological factors [33].

***Anxiety***: Anxiety is a response of the body to a perceived threat which is triggered by an individual’s beliefs, feelings, and thoughts and characterized by worrying thoughts, tension, increased blood pressure, respiratory rate, sweating, pulse rate, dizziness, the difficulty of swallowing, and chest pain [34]. Common anxiety signs and symptoms may include excessive worrying, feeling agitated, restlessness, fatigue, difficulty concentrating, irritability, tense muscles, trouble falling or staying asleep, panic attacks, avoiding social situations, and irrational fears [35]. Factors that are likely to cause anxiety include natural disasters, personal environment such as poverty, early separation from the mother, family conflict, critical and strict parents, personality, family dynamics, brain chemistry, genetic vulnerability.

***Stress***: Stress is a feeling that is initiated when a person perceives that demands exceed resources mobilized by individuals [36]. The symptoms of **s**tress disorder fall under five broad categories: (i) intrusion symptoms, (ii) negative mood, (iii) dissociative symptoms, (iv) avoidance symptoms and (v) arousal symptoms [37]. Factors that may cause stress include natural disasters, the death of a loved one, the threat of death or serious injury, motor vehicle accidents, sexual assault, rape, or domestic abuse, receiving a terminal diagnosis, and surviving a traumatic brain injury [37].

We used the most popular “Depression Anxiety Stress Scales (DASS-21)” to measure the mental health condition (i.e., depression, anxiety and stress status) of our study population. The DASS-21 is a widely used and the most popular measure for assessing the mental health condition (i.e., psychological disorders: depression, anxiety and stress) in adults [38–41]. Historically, the DASS-21 is the shortened version of the DASS-42 developed by Lovibond and Lovibond [38] to assess symptoms of depression, anxiety, and stress among adults. The DASS-21 items are rated on a four-point Likert scale measuring the emotional distress: 0 means “Did not apply to me at all”, 1 means “Applied to me to some degree, or some of the time–Sometimes”, 2 means “Applied to me to a considerable degree or a good part of the time–Often”, and 3 means “Applied to me very much or most of the time–Almost always” [38]. The respondents were asked to think about their experiences in the past seven days and to judge how each statement applied to them. Scores for depression (D), anxiety (A), and stress (S) were calculated by summing the scores for the relevant items. Then the scores on the DASS-21 were multiplied by 2 to calculate the final score. Recommended cut-off scores for conventional severity labels (normal, mild, moderate, severe, extremely severe) are as follows: for normal D: 0–9, A: 0–7, S: 0–14; for mild D: 10–13, A: 8–9; S: 15–18; for moderate D: 14–20, A: 10–14, S: 19–25; for severe D: 21–27, A: 15–19, S: 26–33; and for extremely severe D: 28+, A: 20+, S: 34+ [38]. For the convenience of the analysis, we redefined the three outcome variables depression, anxiety and stress as binary variable assuming the values 0 (zero) for the participants whose scores fall in the normal group, and 1 (one) for those who have any symptom of mental illness (i.e., depression or anxiety or stress).

#### Risk factors / predictor variables

The main predictor variable (i.e., risk factor) of mental health status (depression, anxiety and stress) was the status of whether the household was exposed by riverbank erosion or not (exposed versus non-exposed). In this study, the term ‘exposed’ indicates that the interviewed household had lost any kind of asset by riverbank erosion. In addition to exposure status, the predictor variables included riverbank erosion related, socio-economic, and demographic factors. Riverbank erosion related risk factors consisted of homestead distance from the river (in mile), time lapsed after displacement (internal), own cultivable land (yes/no), amount of cultivable land, loss of cultivable land (yes/no), loss of livestock (yes/no), loss of relatives (yes/no), loss of house (yes/no), social isolation (yes/no), substance abuse (yes/no), social support (yes/no) for rehabilitation, and hope for the land back (yes/no). Demographic factors included gender (male/female), age (in year), family size (number of household members), and number of children. Moreover, the educational level, monthly household income, and occupation of the respondents were considered as socio-economic factors. Displacement is the immediate effect of riverbank erosion. According to the lapsed time of displacement, we classified the respondents into three different groups: non-displaced, displaced within 3 years, and displaced more than 3 years ago. For the convenience of the analysis, respondents’ age was classified into three groups (22–37 years, 38–45 years and 46–80 years), the number of children was categorized into three different classes (1–2, 3–4 and >4), and the housing facilities were classified into three different groups (own house, shelter and rented land).

### Statistical analysis

We summarized the outcome and predictor variables (risk factors) by frequency distribution (number of observations and its percentage). The association between dependent and independent variables was checked using Parson’s chi-squared test. Binary logistic regression analysis was fitted for calculating the odds ratio (OR) along with the 95% confidence interval (CI) to investigate the effects of river erosion and other risk factors on depression, anxiety and stress. The factors found significant in the chi-squared test, were further considered in the logistic regression analysis to quantify and compare their effects on mental health conditions (depression, anxiety and stress). However, to avoid the multicollinearity problem, we excluded some of those significant variables from logistic regression models. Since our main risk factor of interest, exposure status, was defined based on the loss-related risk factors (loss of livestock, loss of house, loss of cultivable land and loss of relatives), we did not include them in our main logistic regression model. Instead, we constructed another logistic regression model with loss-related risk factors as our main risk factors of interest adjusted for others risk factors. For selecting the best model, we followed the stepwise regression procedure of model selection. To evaluate the predictive power/accuracy and goodness of fit of the regression model, we constructed the Receiver Operating Characteristic (ROC) curve and calculated the area under the ROC curve (AUC). Data processing and all the analyses were done using STATA version 16 (StataCorp, College Station, Texas 77845, USA).

### Ethical considerations and participant’s consent

The research protocol was executed with the approval of the Ethical Review Committee of Islamic University, Kushtia-7003, Bangladesh. The questionnaire was translated into native language (Bangla) and read out before the respondents. Being agreed on interview, the educated respondents provided written consent and the uneducated participants put fingerprint signature on the consent form. Informed consent was obtained from each of the participants prior to participation in this study.

## Results

**Fig 3:**
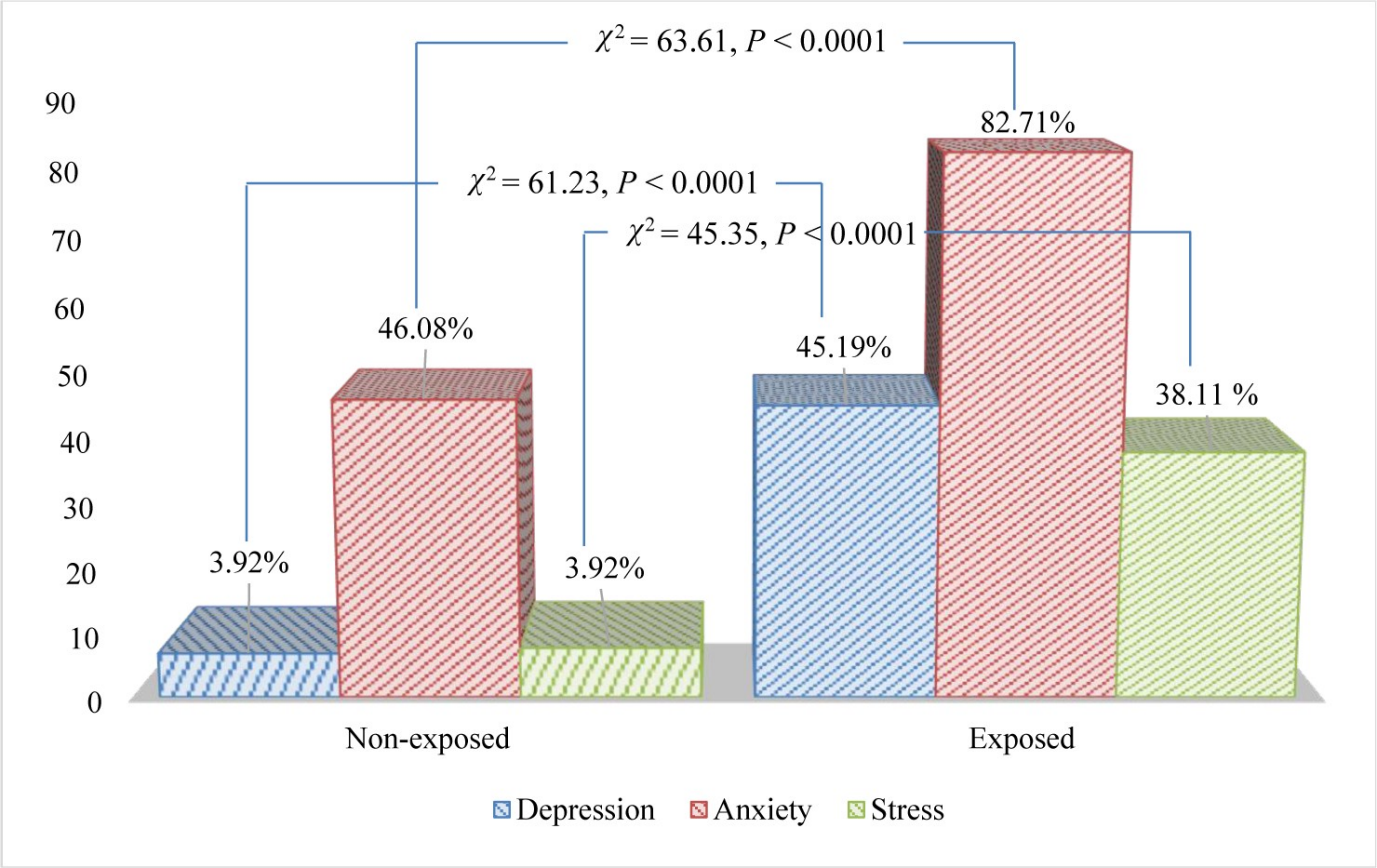
Rate/proportion of depression, anxiety and stress among exposed and non-exposed groups.

Table 1 represents the distribution of respondents’ mental health conditions (depression, anxiety and stress), demographic characteristics, socio-economic status and river erosion-related factors by exposure status (exposed versus non-exposed). Among 611 respondents, 509 (83.31%) were exposed to riverbank erosion, whereas 102 (16.69%) were non-exposed. The overall rates of depression, anxiety and stress were 38.30%, 76.60% and 32.41%, respectively. Table 1 and Fig 3 shows that the prevalence of depression, anxiety and stress was significantly higher among the exposed people compared to their non-exposed counterparts (depression: 45.19% versus 3.92%, *P*<0.001; anxiety: 82.71% versus 46.08%, *P*<0.001; and stress: 38.11% versus 3.92%, *P*<0.001).

**Table 1:** Distribution of mental health, demographic, socio-economic and river erosion related factors by exposure status (exposed and non-exposed) in the study population (N = 611).

		Exposure status	
Characteristic	Overall N = 611 (100%) *n* (%)	Exposed N = 509 (83.31%) *n* (%)	Non-exposed N = 102 (16.69%) *n* (%)
***Mental health related factors***			
**Depression disorder**			
Normal	377 (61.70)	279 (54.81)	98 (96.08)
Symptom present	234 (38.30)	230 (45.19)	4 (3.92)
**Anxiety disorder**			
Normal	143 (23.40)	88 (17.29)	55 (53.92)
Symptom present	468 (76.60)	421 (82.71)	47 (46.08)
**Stress disorder**			
Normal	413 (67.59)	315 (61.89)	98 (96.08)
Symptom present	198 (32.41)	194 (38.11)	4 (3.92)
***Socio-demographic and economic factors***			
**Region**			
Rajbari	410 (67.10)	358 (70.33)	52 (50.98)
Tangail	201 (32.90)	151 (29.67)	50 (49.02)
**Gender**			
Female	317 (51.88)	259 (50.88)	58 (56.86)
Male	294 (48.12)	250 (49.12)	44 (43.14)
**Education**			
Uneducated	280 (45.83)	241 (47.35)	39 (38.24)
Educated	331 (54.17)	268 (52.65)	63 (61.76)
**Age (year)**			
22–37	162 (26.51)	123 (24.17)	39 (38.24)
38–45	216 (35.35)	177 (34.77)	39 (38.24)
46–80	233 (38.13)	209 (41.06)	24 (23.53)
**Number of children**			
1–2	254 (41.57)	206 (40.47)	48 (47.06)
3–4	296 (48.45)	249 (48.92)	47 (46.08)
>4	61 (9.98)	54 (10.61)	7 (6.86)
**Family size**			
<5	249 (40.75)	204 (40.08)	45 (44.12)
5–6	274 (44.84)	227 (44.60)	47 (46.08)
>6	88 (14.40)	78 (15.32)	10 (9.80)
**Monthly Income (taka)**			
<10000	245 (40.10)	207 (40.67)	38 (37.25)
10000–15000	256 (41.90)	213 (41.85)	43 (42.16)
>15000	110 (18.00)	89 (17.49)	21 (20.59)
**Occupation**			
Housewife	265 (43.37)	218 (42.83)	47 (46.08)
Farmer	88 (14.40)	79 (15.52)	9 (8.82)
Business	46 (7.53)	38 (7.47)	8 (7.84)
Day labor	70 (11.46)	62 (12.18)	8 (7.84)
Service	28 (4.58)	23 (4.52)	5 (4.90)
Driver	43 (7.04)	37 (7.27)	6 (5.88)
Others	71 (11.62)	52 (10.22)	19 (18.63)
**Own cultivable land**			
No	490 (80.20)	410 (80.55)	80 (78.43)
Yes	121 (19.80)	99 (19.45)	22 (21.57)
**Amount of cultivable land (decimal)**			
No cultivable land	490 (80.20)	410 (80.55)	80 (78.43)
1–66	84 (13.75)	69 (13.56)	15 (14.71)
67–99	14 (2.29)	13 (2.55)	1 (0.98)
≥100	23 (3.76)	17 (3.34)	6 (5.88)
***River erosion related factors***			
**Internal displacement time (year)**			
Not migrate	238 (38.95)	136 (26.72)	102 (100.00)
≤3	229 (37.48)	229 (44.99)	0 (0.00)
>3	144 (23.57)	144 (28.29)	0 (0.00)
**Homestead distance from river (mile)**			
≤ 0.2	313 (51.23)	266 (52.26)	47 (46.08)
> 0.2	298 (48.77)	243 (47.74)	55 (53.92)
**Loss of livestock**			
No	568 (92.96)	466 (91.55)	102 (100.00)
Yes	43 (7.04)	43 (8.45)	0 (0.00)
**Loss of relatives**			
No	534 (87.40)	432 (84.87)	102 (100.00)
Yes	77 (12.60)	77 (15.13)	0 (0.00)
**Loss of house**			
No	316 (51.72)	214 (42.04)	102 (100.00)
Yes	295 (48.28)	295 (57.96)	0 (0.00)
**Loss of cultivable land**			
No	119 (19.48)	17 (3.34)	102 (100.00)
Yes	492 (80.52)	492 (96.66)	0 (0.00)
**Social isolation**			
No	394 (64.48)	292 (57.37)	102 (100.00)
Yes	217 (35.52)	217 (42.63)	0 (0.00)
**Substance abuse**			
No	588 (96.24)	486 (95.48)	102 (100.00)
Yes	23 (3.76)	23 (4.52)	0 (0.00)
**Social support**			
No	358 (58.59)	256 (50.29)	102 (100.00)
Yes	253 (41.41)	253 (49.71)	0 (0.00)
**Hope for land back**			
No	483 (79.05)	381 (74.85)	102 (100.00)
Yes	128 (21.60)	128 (25.15)	0 (0.00)

The proportion of Rajbari’s respondents were maximum (67.10%) with a higher exposure rate compared to Tangail’s respondents (70.33% versus 50.98%). Among the respondents, the proportions of the female, educated, old aged (>45 years) and housewife respondents were higher with a higher exposure rate compared to their other counterparts. Also, the respondents with 3–4 children, family size 5–6, monthly income 10,000–15,000 taka and no cultivable land were higher in proportion and among them exposure rate was the highest. Unfortunately, most of the households (80.20%) did not have any cultivable land, and among the people having cultivable land (19.80%) the exposure rate was highest for those who had 1 to 66 decimal cultivable lands. It was found that 51.23% of homesteads were within 0.2 miles with higher exposure rate compared to those whose homestead distance was >0.2 miles from riverbank. Among the displaced people, the highest proportion (37.48%) were displaced within three years and these people had higher exposure rate than those whose displacement time is >3 years (44.99% versus 28.29%). Among the respondents, 7.04% lost their live stocks, 12.60% lost their relatives, 48.45% lost their homesteads and 80.52% lost their cultivable land due to the riverbank erosion. Among the exposed respondents, the highest proportion (96.66%) were exposed by losing their cultivable land, followed by the exposure due to losing homesteads/houses (57.96%). We observed that a higher proportion of the exposed people did not isolate themselves from their family (57.37%), did not abuse substances (95.48%), did not need social support (50.29%) and did not hope to get back their lost cultivable land (74.85%).

Table 2 represented the bivariate distribution of outcome variables such as depression, anxiety and stress with other risk factors, and their test (Chi-squared test) of associations. As we saw that the proportions of depression and stress were significantly higher for Rajbari District compared to Tangail District (depression: 42.44% versus 29.85%, *P* = 0.003; and stress: 40.49% versus 15.92%, *P*<0.001), the prevalence of anxiety was similar between the two districts (76.83 versus 76.12, *P* = 0.846). The prevalence of depression, anxiety and stress was higher among the female participants than the males (depression: 43.22% versus 32.99%, anxiety: 78.55% versus 74.49% and stress: 35.96% versus 28.57%) and raised with the increase of age. The respondents who were uneducated, old aged (>45 years), had more than 4 children, had no cultivable land and had lower level income showed the highest rate of depression, anxiety and stress disorder compared to their contrary counterparts. Regarding occupation, the depression rate was the highest among the housewives (44.91%), whereas the rate of anxiety and stress disorder was the highest among the day labourers (anxiety: 82.86% and stress: 38.57%).

**Table 2:** The bivariate distribution of depression, anxiety and stress with different risk factors.

Variables	Depression		*χ*^2^-value (p-value)	Anxiety		*χ*^2^-value (p-value)	Stress		*χ*^2^-value (p-value)
	No N = 377 (61.7%)	Yes N = 234 (38.3%)		No N = 143 (23.4%)	Yes N = 468 (76.6%)		No N = 413 (67.59%)	Yes N = 198 (32.41%)	
	*n* (%)	*n* (%)		*n* (%)	*n* (%)		*n* (%)	*n* (%)	
**Demographic and socio-economic factors**									
**Region**									
Rajbari	236 (57.56)	174 (42.44)	9.0448 (0.003)	95 (23.17)	315 (76.83)	0.0379 (0.846)	244 (59.51)	166 (40.49)	37.1642 (<0.001)
Tangail	141 (70.15)	60 (29.85)		48 (23.88)	153 (76.12)		169 (84.08)	32 (15.92)	
**Gender**									
Female	180 (56.78)	137 (43.22)	6.75 (0.009)	68 (21.45)	249 (78.55)	1.40 (0.236)	203 (64.04)	114 (35.96)	3.80 (0.051)
Male	197 (67.01)	97 (32.99)		75 (25.51)	219 (74.49)		210 (71.43)	84 (28.57)	
**Education level (schooling)**									
Uneducated	161 (42.71)	119 (57.29)	3.86 (0.049)	53 (18.93)	227 (81.07)	5.77 (0.016)	173 (61.79)	107 (38.21)	7.96 (0.005)
Educated	119 (50.85)	115(49.15)		90 (27.19)	241 (72.81)		240 (72.51)	91 (27.49)	
**Age (year)**									
22–37	107 (66.05)	55 (33.95)	3.1 (0.205)	51 (31.48)	111 (68.52)	9.66 (0.008)	116 (71.60)	46 (28.40)	3.15 (0.207)
38–45	136 (62.96)	80 (37.04)		50 (23.15)	166 (76.85)		149 (68.98)	67 (31.02)	
46–80	134 (57.51)	99 (42.49)		42 (18.03)	191 (81.97)		148 (63.52)	85 (36.48)	
**Number of children**									
1–2	169 (66.54)	85 (33.46)	11.91 (0.003)	73 (28.74)	181 (71.26)	9.62 (0.008)	184 (72.44)	70 (27.56)	15.84 (<0.001)
3–4	182 (61.49)	114 (38.51)		63 (21.28)	233 (78.72)		201 (67.91)	95 (32.09)	
>4	26 (42.62)	35 (57.38)		7 (11.48)	54 (88.52)		28 (45.90)	33 (54.10)	
**Family size**									
<5	159 (63.86)	90 (36.14)	1.84 (0.398)	65 (26.10)	184 (73.90)	4.69 (0.096)	170 (68.27)	79 (31.73)	0.17 (0.920)
5–6	161 (58.76)	113 (41.24)		65 (23.72)	209 (76.28)		185 (67.52)	89 (32.48)	
>6	57 (64.77)	31 (35.23)		13 (14.77)	75 (85.23)		58 (65.91)	30 (34.09)	
**Monthly income (Taka)**									
≤10000	146 (59.59)	99 (40.41)	12.45 (0.002)	47 (19.18)	198 (80.82)	6.80 (0.033)	167 (68.16)	78 (31.84)	4.94 (0.085)
10001–15000	147 (57.42)	109 (42.58)		61 (23.83)	195 (76.17)		163 (63.67)	93 (36.33)	
>15000	84 (76.36)	26 (23.64)		35 (31.82)	75 (68.18)		83 (75.45)	27 (24.55)	
**Occupation**									
Housewife	146 (55.09)	119 (44.91)	14.03 (0.029)	60 (22.64)	205 (77.36)	7.75 (0.257)	166 (62.64)	99 (37.36)	12.84 (0.046)
Farmer	57 (64.77)	31 (35.23)		18 (20.45)	70 (79.55)		60 (68.18)	28 (31.82)	
Business	34 (73.91)	12 (26.09)		16 (34.78)	30 (65.22)		34 (73.91)	12 (26.09)	
Day labourer	40 (57.14)	30 (42.86)		12 (17.14)	58 (82.86)		43 (61.43)	27 (38.57)	
Service	18 (64.29)	10 (35.71)		10 (35.71)	18 (64.29)		19 (67.86)	9 (32.14)	
Driver	30 (69.77)	13 (30.23)		10 (23.26)	33 (76.74)		34 (79.07)	9 (20.93)	
Others	52 (73.24)	19 (26.76)		17 (23.94)	54 (76.06)		57 (80.28)	14 (19.72)	
**Own cultivable land**									
No	298 (60.82)	192 (39.18)	0.82 (0.365)	100 (20.41)	390 (79.59)	12.39 (<0.001)	328 (66.94)	162 (33.06)	0.49 (0.486)
Yes	79 (65.29)	42 (34.71)		43 (35.54)	78 (64.46)		85 (70.25)	36 (29.75)	
**Amount of cultivable land**									
No cultivable land	297 (60.86)	191 (39.14)	2.32 (0.508)	100 (20.49)	388 (79.51)	11.85 (0.008)	326 (66.80)	162 (33.20)	1.07 (0.785)
0–66	57 (66.28)	29 (33.72)		31 (36.05)	55 (63.95)		61 (70.93)	25 (29.07)	
67–99	7 (50.00)	7 (50.00)		4 (28.57)	10 (71.43)		9 (64.29)	5 (35.71)	
≥100	16 (69.57)	7 (30.43)		8 (34.78)	15 (65.22)		17 (73.91)	6 (26.09)	
**Riverbank erosion related factors**									
**Time lapsed after internal displacement**									
Not migrate	195 (81.93)	43 (18.07)	104.35 (<0.001)	102 (42.86)	136 (57.14)	94.96 (<0.001)	206 (86.55)	32 (13.45)	91.50 (<0.001)
≤3	84 (36.68)	145 (63.32)		11 (4.80)	218 (95.20)		104 (45.41)	125 (54.59)	
>3	98 (68.06)	46 (31.94)		30 (20.83)	114 (79.17)		103 (71.53)	41 (28.47)	
**Homestead distance (mile)**									
≤0.2	203 (64.86)	110 (35.14)	2.70 (0.100)	67 (21.41)	246 (78.59)	1.43 (0.232)	222 (70.93)	91 (29.07)	3.25 (0.071)
>0.2	174 (58.39)	124 (41.61)		76 (25.50)	222 (74.50)		191 (64.09)	107 (35.91)	
**Loss of livestock**									
No	364 (64.08)	204 (35.92)	19.39 (<0.001)	141 (24.82)	427 (75.18)	9.07 (0.003)	394 (69.37)	174 (30.63)	11.57 (0.001)
Yes	13 (30.23)	30 (69.77)		2 (4.65)	41 (95.35)		19 (44.19)	24 (55.81)	
**Loss relatives**									
No	339 (63.48)	195 (36.52)	5.69 (0.017)	130 (24.34)	404 (75.66)	2.09 (0.148)	373 (69.85)	161 (30.15)	9.85 (0.002)
Yes	38 (49.35)	39 (50.65)		13 (16.88)	64 (83.12)		40 (51.95)	37 (48.05)	
**Loss of house**									
No	245 (77.78)	70 (22.22)	71.11 (<0.001)	121 (38.41)	194 (61.59)	81.70 (<0.001)	257 (81.59)	58 (18.41)	58.12 (<0.001)
Yes	132 (44.59)	164 (55.41)		22 (7.43)	274 (92.57)		156 (52.70)	140 (47.30)	
**Loss of land**									
No	104 (87.39)	15 (12.61)	41.28 (<0.001)	57 (47.90)	62 (52.10)	49.46 (<0.001)	106 (89.08)	13 (10.92)	31.13 (<0.001)
Yes	273 (55.49)	219 (44.51)		86 (17.48)	406 (82.52)		307 (62.40)	185 (37.60)	
**Need separation**									
No	260 (65.66)	136 (34.54)	6.71 (0.006)	118 (29.80)	278 (70.30)	24.49 (<0.001)	277 (69.95)	119 (30.05)	2.46 (0.091)
Yes	117 (54.42)	98 (45.58)		25 (11.63)	190 (88.02)		136 (63.26)	79 (36.74)	
**Substance abuse**									
No	374 (63.61)	214 (36.39)	23.95 (<0.001)	141 (23.98)	447 (76.02)	2.88 (0.089)	408 (69.39)	180 (30.61)	22.94 (<0.001)
Yes	3 (13.04)	20 (86.96)		2 (8.70)	21 (91.30)		5 (21.74)	18 (78.26)	
**Social support**									
No	225 (66.77)	112 (33.23)	8.15 (0.004)	105 (31.16)	232 (68.84)	25.20 (<0.001)	236 (70.03)	101 (29.97)	2.04 (0.154)
Yes	152 (55.47)	122 (44.53)		38 (13.87)	236 (86.13)		177 (64.60)	97 (35.40)	
**Hope for land return**									
No	311 (64.72)	169 (35.28)	8.53 (0.003)	123 (25.62)	357 (74.38)	6.40 (0.013)	329 (68.54)	151 (31.52)	0.92 (0.338)
Yes	66 (50.76)	65 (49.24)		20 (15.15)	112 (84.73)		84 (64.12)	47 (35.61)	

The rates of depression (63.32%), anxiety (95.20%) and stress (54.59%) were significantly higher among the participants who changed their living places within three years compared to their other counterparts. We observed that the prevalence of depression, anxiety and stress was higher among those who lost their livestock (depression: 69.77% versus 35.92%, *P<*0.001; anxiety: 95.35% versus 75.18%, *P* = 0.003; and stress: 55.81% versus 30.63%, *P* = 0.001), relatives (depression: 50.65% versus 36.52%, *P* = 0.017; anxiety: 83.12% versus 75.66%, *P* = 0.148; and stress: 48.05% versus 30.15%, *P* = 0.002), houses (depression: 55.41% versus 22.22%, *P<*0.001; anxiety: 92.57% versus 61.59%, *P*<0.001; and stress: 47.30% versus 18.41%, *P*<0.001) and land (depression: 44.51% versus 12.61%, *P*<0.001; anxiety: 82.52% versus 52.10%, *P*<0.001; and stress: 37.60% versus 10.92%, *P*<0.001) than those who did not lose their relatives, livestock, houses, and land. The people who needed separation from family (depression: 45.58% versus 34.54%, *P* = 0.006; anxiety: 88.02% versus 70.30%, *P*<0.001; and stress: 36.74% versus 30.05%, *P* = 0.091), abused substance (depression: 86.96% versus 36.39%, *P*<0.001; anxiety: 91.30% versus 76.02%, *P* = 0.089; and stress: 78.26% versus 30.61%, *P*<0.001), received social support in terms of borrowing (depression: 44.53% versus 33.23%; anxiety: 86.13% versus 68.84%; and stress: 35.40% versus 29.97%) and had hope for getting back their cultivable land (depression: 49.24% versus 35.28%, anxiety: 84.73% versus 74.38%, and stress: 35.61% versus 31.52%), exhibited higher rate of depression, anxiety and stress disorder in the river erosion prone areas.

Table 3 represents the results [odds ratio (OR) and its 95% confidence interval (CI)] of logistic regression analysis. The binary logistic regression analysis revealed that the exposed participants were 8.72 times, 2.35 times, and 5.17 times more likely to be depressed, anxious, and stressed, respectively, than the non-exposed participants (OR_D_ = 8.72, 95% CI = 2.90–26.26; OR_A_ = 2.35, 95% CI = 1.37–4.05; OR_S_ = 5.17, 95% CI = 1.66–16.08). Logistic regression models, where loss-related risk factors were our main interest (S1 Table), revealed that the odds of depression, anxiety and stress were 2.69, 3.13 and 1.98 times higher among the people who lost their livestock than those who did not lose their livestock (OR_D_ = 2.69, 95% CI = 1.25–5.81; ORA = 3.13, 95% CI = 1.70–13.93; OR_S_ = 1.98, 95% CI = 1.14–4.14). Similarly, those who lost their cultivable land had almost 2-fold higher likelihood of depression, anxiety and stress (OR_D_ = 2.28, 95% CI = 1.14–4.59; OR_A_ = 1.82, 95% CI = 1.09–3.06; OR_S_ = 1.86, 95% CI = 1.09–3.99). Participants who lost their homestead were respectively 2.08, 2.79 and 2.18 times more likely to have depression, anxiety and stress than those who did not lose their homestead (OR_D_ = 2.08, 95% CI = 1.15–3.75; OR_A_ = 2.79, 95% CI = 1.38–5.63; OR_S_ = 2.18, 95% CI = 1.20–3.93). The people who lost their relatives had 1.79 times higher odds to be stressed than those who did not lost their relatives (OR_S_ = 1.79, 95% CI = 1.03–3.18).

**Table 3:** Logistic regression final model showing the adjusted odds ratio of predictors of DAS.

Characteristic	Outcome		
	Depression OR_D_ (95% CI)	Anxiety OR_A_ (95% CI)	Stress OR_S_ (95% CI)
**Exposed**			
No	Reference	Reference	Reference
Yes	8.72 (2.90–26.26)***	2.35 (1.37–4.05)**	5.17 (1.66–16.08)**
**Region**			
Tangail	Reference		Reference
Rajbari	2.35 (1.42–3.91)***		6.84 (3.82–12.25)***
**Gender**			
Male	Reference	Reference	Reference
Female	2.22 (1.49–3.31)***	1.67 (1.06–2.62)*	2.07 (1.36–3.14)***
**Educational status**			
Educated			Reference
Uneducated			1.53 (1.01–2.33)*
**Age (Year)**			
22–37		Reference	
38–45		1.71 (1.01–2.88)*	
46–80		1.97 (1.12–3.47)*	
**Number of children**			
1–2	Reference		Reference
3–4	0.99 (0.65–1.51)		0.85 (0.54–1.33)
>4	2.07 (1.04–4.13)*		2.00 (1.12–4.05)*
**Monthly income (Taka)**			
>15000	Reference	Reference	
≤10000	2.19 (1.20–3.99)*	1.76 (1.02–3.13)*	
10001–15000	2.61 (1.45–4.70)***	1.37 (0.79–2.39)	
**Own cultivable land**			
Yes		Reference	
No		1.79 (1.10–2.93)*	
**Time lapsed after displacement**			
Not displaced	Reference	Reference	Reference
≤3 years	4.68 (2.81–7.80)***	9.22 (4.52–18.82)***	6.27 (3.52–11.15)***
>3 years	0.99 (0.57–1.73)	1.07 (0.85–3.11)	1.31 (0.71–2.39)
**Homestead distance (Mile)**			
≤0.2	Reference		Reference
>0.2	1.80 (1.21–2.69)**		2.09 (1.37–3.21)***
**Substance abuse**			
No	Reference		Reference
Yes	8.25 (2.18–31.20)		4.71 (1.49–14.88)**
**Hope for land return**			
No	Reference		Reference
Yes	1.83 (1.12–3.00)*		1.74 (1.02–2.95)*
**AUC**	**0.82**	**0.80**	**0.82**

*P < 0.05

**P < 0.01

***P < 0.001.

OR_D_: Odds ratio for depression, OR_A_: Odds ratio for anxiety, OR_S_: Odds ratio for stress, CI: Confidence interval, AUC: Area under the curve.

In Rajbari District, participants were 2.35 and 6.84 times more likely to be depressed and stressed than Tangail’s participants (OR_D_ = 2.35, 95% CI = 1.42–3.91; OR_S_ = 6.84, 95% CI = 3.82–12.25). However, female participants were respectively 2.22, 1.67 and 2.07 times more likely to be depressed, anxious, stressed than males (OR_D_ = 2.22, 95% CI = 1.49–3.31; OR_A_ = 1.67, 95% CI = 1.06–2.62; OR_S_ = 2.07, 95% CI = 1.36–3.14). Although respondent’s age did not have any significant effect on depression and stress, the odds of anxiety was almost 2-fold higher among the middle-aged (38–45 years) (OR_A_ = 1.71, 95% CI = 1.01–2.88) and oldest-aged (>45 years) (OR_A_ = 1.97, 95% CI = 1.12–3.47) respondents than their young-aged (≤37 years) counterparts. The people having more than four children were 2 times more likely to be depressive and stressed than those who had 1–2 children (OR_D_ = 2.07, 95% CI = 1.04–4.13; OR_S_ = 2.00, 95% CI = 1.12–4.05). The respondents with monthly income <10,000 taka and 10,001–15,000 taka respectively exhibited 2-fold and 3-fold higher odds of depression compared to those having monthly income >15,000 taka (OR_D_ = 2.19, 95% CI = 1.20–3.99; OR_D_ = 2.61, 95% CI = 1.45–4.70). The people having no cultivable land was 79% higher likelihood of anxiety than those who have cultivable land (OR_A_ = 1.79, 95% CI = 1.10–2.93). Among the displaced participants, those who were displaced within 3 years were respectively 4.68, 9.22, and 6.27 times more likely to have depression, anxiety, and stress than those who were not displaced (OR_D_ = 4.68, 95% CI = 2.81–7.80; OR_A_ = 9.22, 95% CI = 4.52–18.82; OR_S_ = 6.27, 95% CI = 3.52–11.15). Unexpectedly, the likelihoods of depression and stress were almost 2-fold higher for the participants whose houses were located more than 0.2 miles from the riverbank than those who lived within 0.2 miles (OR_D_ = 1.80, 95% CI = 1.21–2.69; OR_S_ = 2.09, 95% CI = 1.37–3.21). The odds of depression and stress were respectively 8.25 and 4.71 times larger for the respondents who abused substances than those who did not abuse substances (OR_D_ = 8.25, 95% CI = 2.18–31.20; OR_S_ = 4.71 95% CI = 1.49–14.88). The people who had hope for the return of their lost cultivable land had almost 2-fold higher odds of depression and stress than those who didn’t hope (OR_D_ = 1.83, 95% CI = 1.12–3.00; OR_S_ = 1.74, 95% CI = 1.02–2.95). The areas under the ROC curves for depression, anxiety, and stress were 0.82, 0.80, and 0.82, respectively; which indicates that the regression models were fitted well.

## Discussion

We delved into the literature regarding the effect of river erosion on mental health, but there was a scarcity of studies directly related to the issue in the study areas or country. However, for the sake of comparison and discussion, the effect of some other disasters like floods could be considered anyway. Our study revealed that the prevalence of depression, anxiety and stress (DAS) was significantly higher among the people who were exposed to river erosion than those who were non-exposed (Fig 3), which was consistent with the findings of previous studies conducted in Bangladesh [6] and in other countries [42–45]. Being consistent with Arobi et al. [6], Pooja and Nagalakshami [46] and Thomas et al. [28], multiple logistic regression analysis also confirmed that the people who were exposed to river erosion were more likely to develop DAS disorder compared to non-exposed participants. The reason was that the participants who were exposed, lost various kinds of assets (such as house, land, livestock, etc.), and took narcotics to remove their stress/tension/frustration. Moreover, river erosion exposure could have driven pre-existing psychological distress, sleep problems, somatic complaints, and psychosocial, behavioral problems [4, 7]. We found that the rate of depression and stress significantly varied between the two selected districts. The rate of depression and stress were significantly higher among the people of Rajbari District compared to those from Tangail District. One of the possible causes could be that the riverbeds of the Brahmaputra along the Tangail District were used for agricultural production in the dry season, and the people affected by erosion could get access to the beds according to their old land demarcations. Unfortunately, our survey confirmed that the people affected by the Ganges erosion along the Rajbari District did not have such kind of opportunity may be due to the local soil quality and hydraulic patterns.

As already discussed in some existing studies [10, 46–49], we found that the odds of DAS disorder was higher among the females compared to their male counterparts. The prevalence of DAS was significantly higher among the uneducated people than the educated people, which is consistent with the findings of the study conducted by Weyerer et al. [49]. However, Asghari et al. [47] showed that mental condition does not depend on the level of education. The DAS rates were higher in the older age group. The people between 38–45 and >45 years of age were more likely to be anxious than those of ≤37 years. A similar discussion was also found in some existing studies [10, 49–52]. We observed that the higher the number of children, the higher the rate of DAS among the study population. It might be due to the fact that the more the children the more the economic burden is induced by river erosion. The respondents having more children had higher odds of developing stress disorder compared to those who had less number of children as it was very difficult for the affected people to manage a big sized family in financial consideration. However, Norizan and Shamsuddin [53] found that stress does not depend on the number of children of the respondents.

The richer respondents (having monthly income >15,000 Taka) were less likely to be depressed compared to the poor respondents (monthly income ≤10,000 Taka) as the higher income group had more opportunities to cope with the shock. The depression rate was the highest among housewives whereas the anxiety and stress rates were the highest among the day labourers. The odds of being anxious was higher among the landless participants compared to those who owned land. Internal displacement was a vital factor for depression, anxiety and stress. We observed that the rate of DAS was significantly higher among the people who were displaced internally due to river erosion compared to those who were not displaced. The result with the displacement was consistent with the findings of previous studies conducted in Norway among the refugees [54] and in the United Kingdom among the flood-affected individuals [42]. The odds of developing depression and stress was significantly smaller among the participants whose houses were within 0.2 miles of the riverbank than those whose houses were more than 0.2 miles away. We suspected that nearer households had more access to the river centric economic activities like dry-season-agriculture in riverbed, fishing, boating, and received more support from various kind of relief and social safety net from both government and non-government organizations. The majority of the participants living within 0.2 miles of the riverbank were non-displaced, or they were displaced more than three years ago. The respondents who were displaced within 3 years were more likely to experience DAS disorder compared to those who were not displaced. Similar types of results were found in some previous studies [42–45]. Recently displaced people, on the one hand, could not forget their terrible experience and manage time to adapt to the burden of disaster, on the other hand, they were also out of governmental tracking for financial support or social safety net programs; so they felt more depressed.

The depression and stress levels were respectively 3-fold and 2-fold higher among those who lost livestock by river erosion. In Bangladesh, livestock (cow, buffalo, goat, sheep, etc.) were the main assets and wealth for the low-earning riparian people. After losing their livestock they were more helpless and got more depressed and anxious. The respondents who lost their homestead by river erosion were more likely to get depressed, anxious and stressed than those who did not lose their homestead as they had to spend their savings for re-placing and re-building the destroyed houses. Depression, anxiety and stress were highly prevalent among the people who abused substances. The affected people abused different types of substances to forget their miseries due to river erosion. In this regard,Martín-Merino et al. [55] similarly identified that anxiety was associated with heavy smoking, alcohol use, and addiction problems, as well as stress, sleep, and depression disorders.

### Limitations of the study

There were some limitations of this study, which could be considered in interpreting the study results. Firstly, this study was a cross-sectional study with only two river erosion-prone areas (Rajbari and Tangail Districts) and a moderate number of participants (N = 611) that prevented us to draw a generalized scenario commit it as the general scenario of the country. Secondly, we limited our study to only the three major mental health problems: depression, anxiety and stress disorder as the representative of the mental health status among the study population. However, there were some other common mental health problems/disorders (such as neurodevelopmental disorders, sleep-wake disorders, schizophrenia spectrum disorder, psychiatric disorders, psychotic disorder, bipolar and related disorders, etc.) among the study population that were not considered in this study.

## Conclusion

In our study, we found that the people who were exposed to riverbank erosion were more likely to develop DAS disorder and experience worse mental conditions compared to their non-exposed counterparts. We also observed that the odds of mental illness problem was higher among females or housewives, older, uneducated, poor and had ≥4 children compared to their respective counterparts.

Riverbank erosion is a regular phenomenon, occurs due to a wreath of anthropogenic and natural reasons. Van Tho [56] discussed many solutions can be applied by governments to cope with river erosion. Though the government was sometimes successful to protect some important structures or towns from river erosion by building embankments on the banks, these cannot be a sustainable or comprehensive solution to the distress for all the affected people across the country. In the wide range, these direct initiatives were often turned into a huge waste of public fund. Moving people from erosion-prone areas to safe places could have been a priority to protect people’s lives and property, but Bangladesh is one of the most densely populated countries in the world.

So, as in Mutton and Haque [57], we also focused on a range of socio-demographic and socio-economic variables in determining the coping ability and reducing mental health illness. Since the financially better-off-households were found to shoulder less mental health disorder in our result, the government could make some policy efforts or initiatives like transferring financial assistance (as social safety net program) directly to the people exposed to river erosion. Our findings concluded that the government should shed special light on females or housewives, older, uneducated, poor and larger family compared to their respective counterparts to reduce gaps in the burden of mental health illness due to river erosion. Educating people is an important approach to reduce mental illness. The internally displaced people should also be carefully included in the assistance program as the recently displaced people settled away from the banks and were thus outside the purview of the government for social safety net and rehabilitation programs. Different types of loan related schemes should be initiated at low-interest rate in the affected areas.

Having access to the reclaimed land for agricultural activities, might be one of the reasons in Tangail District to have less depression and stress than in Rajbari, though the erosion pattern in the former area was more striking and recent than the latter. So the management and redistribution process of the reclaimed land among the affected people, especially in the dry season for agricultural purposes, should be fair and transparent controlled by local and central governments. To improve the mental health services in Bangladesh, further well-designed epidemiological and clinical studies are needed in river erosion prone areas.

## Supporting information

S1 Appendix Study materials (Questionnaire). (DOCX)

S1 Table Logistic regression final model (i.e., best model) showing the adjusted odds ratio of predictors of DAS. (DOCX)
