# Supplementary material for: Effects of riverbank erosion on mental health of the affected people in Bangladesh
Source: PLoS One. 2021 Jul 22;16(7):e0254782. doi: 10.1371/journal.pone.0254782 (PMC8297774; doi:10.1371/journal.pone.0254782)
Supplement: S1 Appendix — (DOCX) [file pone.0254782.s001.docx]

**S1 Appendix. Study materials (Questionnaire)**

The questionnaires consist of three sections as follows. **Section A** consists of ten socio-demographic and economic factors, including gender, age, education level, number of children, family size, cultivable land, occupation, and monthly household income. **Section B** includes twelve riverbank erosion-related factors such as exposed/non-exposed, internal displacement (yes/no), homestead distance, loss of house, loss of cultivable land, loss of a relative, loss of livestock, social isolation, substance abuse, and social support.

The questionnaire in **Section C** is designed to explore the psychological characteristics of the respondents and consisted of twenty-one questions on depression, anxiety, and stress scale (DASS-21). There were 21 items in this scale. DASS-21 is a set of three self-report scales designed to measure the emotional states of depression, anxiety, and stress. Each of the three DASS-21 scales contains 7 items, divided into subscales with similar content. In other words, it measures emotional distress in three 7-item dimensions: 1^st^ depression (e.g., “I found it difficult to work up the initiative to do things”); 2^nd^ anxiety (e.g., “I experienced breathing difficult”); 3^rd^ stress (e.g., “I found it hard to wind down”). The questionnaire containing all these questions is shown below.

| **Identification Information** | | | | | | | | |
| --- | --- | --- | --- | --- | --- | --- | --- | --- |
| 1. | Name of respondent:_ _ _ _ _ _ _ _ _ _ _ _ _ _ _ _ _ _ | | | | | | | |
| 2. | Father’s name: …………………………………………. | | | | | | | |
| 3. | Mother’s name:_ _ _ _ _ _ _ _ _ _ _ _ _ _ _ _ _ _ _ _ _ | | | | | | | |
| 4. | Age of respondent (in completed years):______________ | | | | | | | |
| 5. | Date of birth (Day/Month/Year): | | | | | | | |
| 6. | Gender of respondent: | - Male | | - Female | | | |  |
| 7. | NID number: | | | | | | | |
| 8. | Name of village:_ _ _ _ _ _ _ _ _ _ _ _ _ _ _ _ | | | | | | | |
| 9. | Name of the union :­ ­_ _ _ _ _ _ _ _ _ _ _ _ _ _ _ _ | | | | | | | |
| 10. | Was consent given by the respondent? | | - Yes | | | - No | | |
| 11. | Mobile number of respondent: | | | | | | | |
| **Section A: Socio-demographic and Economic information** | | | | | | | | |
| 1. | Gender of the respondent | | | | - Male | | - Female | |
| 2. | Education level of the respondent | | | |  | |  | |
| 3. | Age of the respondent | | | |  | |  | |
| 4. | Marital status of the respondent | | | |  | |  | |
| 5. | How many people have been living in your household for the last 6 months? | | | |  | |  | |
| 6. | How many children you have? | | | |  | |  | |
| 7. | Occupation of the respondent | | | |  | |  | |
| 8. | Monthly household income | | | |  | |  | |
| 9. | Do you have cultivable land of your own? | | | | - Yes | | - No | |
| 10. | If yes, the amount of cultivable land | | | |  | |  | |
| **Section B: Riverbank erosion related information** | | | | | | | | |
| 11. | Were you ever exposed to river erosion? | | | | - Yes | | - No | |
| 12. | Did you have to change your living place | | | | - Yes | | - No | |
| 13. | If yes, within how many months you left the place | | | |  | |  | |
| 14. | Household distance from the river | | | |  | |  | |
| 15. | Did you lose cultivable land? | | | | - Yes | | - No | |
| 16. | Did you lose any relative due to river erosion? | | | | - Yes | | - No | |
| 17. | Did you lose any livestock due to river erosion? | | | | - Yes | | - No | |
| 18. | Did you have to separate from your family members or children for that event? | | | | - Yes | | - No | |
| 19. | Did you try to cope with your grief or frustration by substance use? | | | | - Yes | | - No | |
| 20. | Did you get social support from newly displacement area? | | | | - Yes | | - No | |
| 21. | Do you have any hope that you will get back your field/cultivable land? | | | | - Yes | | - No | |

| **Section C: Depression, Anxiety and Stress Scale - 21 Items (DASS-21)** | | | | | |
| --- | --- | --- | --- | --- | --- |
| 1 (s) | I found it hard to wind down | 0 | 1 | 2 | 3 |
| 2 (a) | I was aware of dryness of my mouth | 0 | 1 | 2 | 3 |
| 3 (d) | I couldn’t seem to experience any positive feeling at all | 0 | 1 | 2 | 3 |
| 4 (a) | I experienced breathing difficulty (e.g. excessively rapid breathing, breathlessness in the absence of physical exertion) | 0 | 1 | 2 | 3 |
| 5 (d) | I found it difficult to work up the initiative to do things | 0 | 1 | 2 | 3 |
| 6 (s) | I tended to over-react to situations | 0 | 1 | 2 | 3 |
| 7 (a) | I experienced trembling (e.g. in the hands) | 0 | 1 | 2 | 3 |
| 8 (s) | I felt that I was using a lot of nervous energy | 0 | 1 | 2 | 3 |
| 9 (a) | I was worried about situations in which I might panic and make a fool of myself | 0 | 1 | 2 | 3 |
| 10 (d) | I felt that I had nothing to look forward to | 0 | 1 | 2 | 3 |
| 11 (s) | I found myself getting agitated | 0 | 1 | 2 | 3 |
| 12 (s) | I found it difficult to relax | 0 | 1 | 2 | 3 |
| 13 (d) | I felt down-hearted and blue | 0 | 1 | 2 | 3 |
| 14 (s) | I was intolerant of anything that kept me from getting on with what I was doing | 0 | 1 | 2 | 3 |
| 15 (a) | I felt I was close to panic | 0 | 1 | 2 | 3 |
| 16 (d) | I was unable to become enthusiastic about anything | 0 | 1 | 2 | 3 |
| 17 (d) | I felt I wasn’t worth much as a person | 0 | 1 | 2 | 3 |
| 18 (s) | I felt that I was rather touchy | 0 | 1 | 2 | 3 |
| 19 (a) | I was aware of the action of my heart in the absence of physical exertion (e.g. sense of heart rate increase, heart missing a beat) | 0 | 1 | 2 | 3 |
| 20 (a) | I felt scared without any good reason | 0 | 1 | 2 | 3 |
| 21 (d) | I felt that life was meaningless | 0 | 1 | 2 | 3 |
| 0 = Did not apply to me at all,  1 = Applied to me to some degree, or some of the time 2 = Applied to me to a considerable degree or a good part of time 3 = Applied to me very much or most of the time | | | | | |
