# Supplementary material for: Effects of riverbank erosion on mental health of the affected people in Bangladesh
Source: PLoS One. 2021 Jul 22;16(7):e0254782. doi: 10.1371/journal.pone.0254782 (PMC8297774; doi:10.1371/journal.pone.0254782)
Supplement: S1 Table — (DOCX) [file pone.0254782.s002.docx]

**S1 Table.** Adjusted odds ratios (ORs) for the loss-related predictors (loss of livestock, land, house and relatives) adjusting for other predictors in a final logistic regression model (i.e., the best model including only the significant variables)

| **Characteristic** | **Outcome** | | |
| --- | --- | --- | --- |
|  | **Depression**  **OR_D_ (95% CI)** | **Anxiety**  **OR_A_ (95% CI)** | **Stress**  **OR_S_ (95% CI)** |
| **Loss of livestock** |  |  |  |
| No | Reference | Reference | Reference |
| Yes | 2.69 (1.25–5.81)* | 3.13 (1.70–13.93)** | 1.98 (1.14–4.14)* |
| **Loss of land** |  |  |  |
| No | Reference | Reference | Reference |
| Yes | 2.28 (1.14–4.59)* | 1.82 (1.09–3.06)* | 1.86 (1.09–3.99)* |
| **Loss of house** |  |  |  |
| No | Reference | Reference | Reference |
| Yes | 2.08 (1.15–3.75)* | 2.79 (1.38–5.63)** | 2.18 (1.20–3.93)* |
| **Loss of relatives** |  |  |  |
| No | Reference | Reference | Reference |
| Yes | 1.36 (0.76–2.43) | 1.11 (0.54–2.25) | 1.79 (1.03–3.18)* |
| **Region** |  |  |  |
| Tangail | Reference |  | Reference |
| Rajbari | 2.92 (1.74–4.91)*** |  | 8.64 (4.71–15.86)*** |
| **Gender** |  |  |  |
| Male | Reference | Reference | Reference |
| Female | 2.31 (1.54–3.46)*** | 1.73 (1.10–2.72)* | 2.14 (1.41–3.27)*** |
| **Educational status** |  |  |  |
| Educated |  |  | Reference |
| Uneducated |  |  | 1.53 (1.02–2.34)* |
| **Age (Year)** |  |  |  |
| ≤37 |  | Reference |  |
| 38–45 |  | 1.77 (1.04–3.01)* |  |
| >45 |  | 2.07 (1.16–3.67)* |  |
| **Number of children** |  |  |  |
| 1–2 | Reference |  | Reference |
| 3–4 | 0.98 (0.64–1.49) |  | 0.83 (0.53–1.31) |
| >4 | 1.99 (1.04–3.95)* |  | 1.98 (1.01–4.04)* |
| **Monthly income (Taka)** |  |  |  |
| >15000 | Reference | Reference |  |
| ≤10000 | 1.98 (1.08–3.63)* | 1.62 (0.91–2.88) |  |
| 10001–15000 | 2.46 (1.36–4.44)** | 1.37 (0.79–2.39) |  |
| **Own cultivable land** |  |  |  |
| Yes |  | Reference |  |
| No |  | 1.71 (1.04–2.79)* |  |
| **Time lapsed after displacement** |  |  |  |
| Not displaced | Reference | Reference | Reference |
| ≤3 years | 3.23 (1.60–6.51)*** | 4.50 (1.91–10.61)*** | 4.19 (2.00–8.76)*** |
| >3 years | 0.74 (0.37–1.50) | 1.06 (0.55–2.07) | 0.92 (0.44–1.92) |
| **Homestead distance (Mile)** |  |  |  |
| ≤0.2 | Reference |  | Reference |
| >0.2 | 1.81 (1.21–2.72)** |  | 2.15 (1.39–3.33)*** |
| **Substance abuse** |  |  |  |
| No | Reference |  | Reference |
| Yes | 7.56 (1.93–29.57)** |  | 3.87 (1.21–12.39)* |
| **Hope for land return** |  |  |  |
| No | Reference |  | Reference |
| Yes | 2.03 (1.23–3.36)** |  | 1.79 (1.04–3.09)* |
| **AUC** | **0.82** | **0.81** | **0.83** |

**P* < 0.05, ***P* < 0.01, ****P* < 0.001.

OR_D_: Odds ratio for depression, OR_A_: Odds ratio for anxiety, OR_S_: Odds ratio for stress, CI: Confidence interval, AUC: Area under the curve.
